# Supplementary material for: Analysis of PPARγ Signaling Activity in Psoriasis
Source: Int J Mol Sci. 2021 Aug 10;22(16):8603. doi: 10.3390/ijms22168603 (PMC8395241; doi:10.3390/ijms22168603)
Supplement: Supplementary file 1 [file ijms-22-08603-s001.zip › Supplemental materials_Analysis of PPARg signaling activity in psoriasis/Pathway models/Models images and html files/Interleukin-17 and interleukin-22 signaling in psoriasis/11164.html]

MAP3K7


# Protein MAP3K7

|  |  |
| --- | --- |
| URN | urn:agi-llid:6885 |
| Total Entities | 0 |
| Connectivity | 2267 |
| Name | MAP3K7 |
| Description | mitogen-activated protein kinase kinase kinase 7 |
| Notes | The protein encoded by this gene is a member of the serine/threonine protein kinase family. This kinase mediates the signaling transduction induced by TGF beta and morphogenetic protein (BMP), and controls a variety of cell functions including transcription regulation and apoptosis. In response to IL-1, this protein forms a kinase complex including TRAF6, MAP3K7P1/TAB1 and MAP3K7P2/TAB2; this complex is required for the activation of nuclear factor kappa B. This kinase can also activate MAPK8/JNK, MAP2K4/MKK4, and thus plays a role in the cell response to environmental stresses. Four alternatively spliced transcript variants encoding distinct isoforms have been reported. [provided by RefSeq, Jul 2008] |
| Primary Cell Localization | Cytoplasm |
| Class | Protein kinase |
| ObjectType | Protein |

---

|  |  |
| --- | --- |
| Pathway | Osteoarthritis |
|  | Fatty Acids Block TLR Signaling in Enteroendocrine Cell |
|  | Toll-like Receptors Act through MYD88-TIRAP Signaling |
|  | NOD-like Receptors |
|  | Antiviral Signaling through Pattern Recognition Receptors |
|  | Toll-like Receptors Act through MYD88 Signaling |
|  | TLR4 Signaling in Leukocytes |
|  | T-Cell Dependent B-Cell Activation |
|  | T-Cell Independent B-Cell Activation |
|  | V(D)J Recombination Activation |
|  | Immunoglobulin Class-Switch Recombination Activation |
|  | Peripheral T-Cell Tolerance |
|  | CD8+ T-Cell Activation |
|  | Th1-Cell Differentiation |
|  | Treg-Cell Differentiation |
|  | Th2-Cell Differentiation |
|  | T-Cell Receptor Signaling |
|  | Th17-Cell Differentiation |
|  | Toll-like Receptors in Sterile Inflammation |
|  | Toll-like Receptor Independent Sterile Inflammation |
|  | Prostaglandin E2 Receptor Signaling in Neurons |
|  | Basophil Activation |
|  | Eosinophil Survival by Cytokine Signaling |
|  | Mast-Cell Activation without Degranulation through TNFSF8 Signaling |
|  | Mast-Cell Activation without Degranulation |
|  | Mast-Cell Activation without Degranulation through IL33/IL1RL1 Signaling |
|  | Mast-Cell Activation without Degranulation through IL1R1 and TLR Signaling |
|  | Macrophage M1 Lineage |
|  | CD16/CD14 Proinflammatory Monocyte Activation |
|  | AXL Receptor Inhibits Macrophages and Dendritic Cells Function |
|  | Neutrophil Recruitment and Priming |
|  | Negative Acute Phase Proteins Synthesis |
|  | Positive Acute Phase Proteins Synthesis |
|  | Vascular Endothelial Cell Activation by Cytokines |
|  | Adipokines Production by Adipocyte |
|  | IL1B Expression Targets -> Nociception |
|  | Apoptosis |
|  | Scavenger Receptor OLR1 in Inflammation Related Endothelial Dysfunction |
|  | Dendritic Cells Function in Atherosclerosis |
|  | RAGE/AGER and S100 Proteins in Cardiovascular Injury |
|  | Th1-Cells Activation and Proliferation in Atherosclerosis |
|  | Dilated Cardiomyopathy |
|  | Myocardial Infarction |
|  | MAVS in Antiviral Innate Immune Response in Myocarditis |
|  | Lymphocyte Mediated Myocardial Injury in Myocarditis |
|  | Toll-like Receptors (TLR) in Antiviral Innate Immune Response |
|  | Inflammatory Reaction in Acne Vulgaris |
|  | Keratinocyte Proliferation in Acne Vulgaris |
|  | Atopic Dermatitis Onset |
|  | Mast-Cells Activation in Atopic Dermatitis |
|  | WNT Signaling in Melanoma |
|  | IL17 Signaling in Psoriasis |
|  | Proteins Involved in Psoriasis |
|  | Dendritic Cells Function in Psoriasis |
|  | Apoptotic Keratinocytes Clearance Recession in Systemic Lupus Erythematosus |
|  | Dendritic Cell Activation in Systemic Lupus Erythematosis |
|  | Th17-Cell Function in Systemic Lupus Erythematosus |
|  | B-Cell Activation in Systemic Lupus Erythematosus |
|  | Th2-Cell Function in Systemic Lupus Erythematosus |
|  | Acute Cytotoxic CD8+T-Cell Response against Melanocytes |
|  | T-Cell Cytotoxic Response against Melanocytes in Vitiligo |
|  | Humoral Immunity in Vitiligo |
|  | Treg-Cell Function in Diabetes Mellitus Type 1 |
|  | Natural Killer T-Cell Roles in Diabetes Mellitus Type 1 |
|  | non-Suppressive Treg-Cell in Diabetes Mellitus Type 1 |
|  | Plasmacytoid Dendritic Cell in Diabetes Mellitus Type 1 |
|  | TLR2 Signaling in Treg-Cell in Type 1 Diabetes (Animal Model) |
|  | Toll-like Receptors in beta-Cell |
|  | Peripheral Tolerance to Autoantigens Recession in Diabetes Mellitus Type 1 |
|  | TLR7 and TLR9 in Plasmacytoid Dendritic Cell |
|  | Conventional Dendritic Cell Role in Diabetes Mellitus Type 1 |
|  | Treg-Cell Activation in Diabetes Mellitus |
|  | Adiponectin in Insulin Resistance Prevention |
|  | Insulin Resistance in Myocytes Induced by Obesity |
|  | Adipose Tissue Inflammation Provokes Insulin Resistance |
|  | Hepatic Stellate Cells in non-Alcoholic Fatty Liver Disease |
|  | beta-Cell Destruction through Cytokines in Diabetes Mellitus |
|  | beta-Cell Destruction in Diabetes Mellitus Type 1 |
|  | Adipokines Production by Adipocyte Impaired in Obesity |
|  | CD4+T-Cell Response in Celiac Disease |
|  | CD8+ T-Cell Response in Celiac Disease |
|  | Celiac Disease |
|  | Crohn's Disease |
|  | Macrophage Dysfunction |
|  | Dendritic Cell Dysfunction in Crohn's disease |
|  | CagA Phosphorylation Independent Signaling |
|  | Dendritic Cell Function in Ulcerative Colitis |
|  | Epithelial Cell in the Innate Immune Response in Ulcerative Colitis |
|  | Nicotine anti-Inflammatory Effect in Ulcerative Colitis |
|  | Th2-Cell Function in Ulcerative Colitis |
|  | T-Cell Cytotoxic Response in Ulcerative Colitis |
|  | Antiphospholipid Antibodies in Endothelial Cell |
|  | B-Cell Chronic Lymphocytic Leukemia |
|  | Diffuse Large-B-Cell Lymphoma |
|  | Diffuse Large-B-Cell Lymphoma ABC Subtype |
|  | Follicular Lymphoma |
|  | Hodgkin Lymphoma |
|  | Mantle Cell Lymphoma |
|  | mTOR/NF-kB/BCR Signaling Disregulation in Mantle Cell Lymphoma |
|  | Mucosa-Associated Lymphoid Tissue (MALT) Lymphoma |
|  | Splenic Marginal Zone Lymphoma |
|  | TNFR/NF-kB Classical Pathway in Multiple Myeloma Plasma Cells |
|  | Osteoclast Activation in Multiple Myeloma |
|  | Multiple Myeloma |
|  | Antiphospholipid Antibodies and Endothelial Cell Interaction |
|  | T-Cell Acute Lymphoblastic Leukemia |
|  | Il17 Signaling Related Neutrophilia in Asthma |
|  | IL33 Signaling Related Eosinophilia in Asthma |
|  | TSLP Signaling in Bronchial Epithelial Cell in Asthma |
|  | IL17F Signaling in Bronchial Epithelial Cell in Asthma |
|  | Goblet-Cell Related Mucus Secretion in Asthma |
|  | Club-Cell Role in Asthma |
|  | Basophil Activation in Asthma |
|  | Antigen-Presenting Cell Role in Asthma |
|  | B-Cell Mediated IgE Production in Asthma |
|  | Th17-Cell Differentiation in Asthma |
|  | Th2-Cell Response in Asthma |
|  | Mucin Production in Goblet Airway Epithelial Cells |
|  | CFTR Facilitates Oxidative Stress in Airway Epithelium in CF |
|  | Airway Epithelial Cell Dysfunction |
|  | CFTR Expression in Epithelial Cells (Class I Mutations) |
|  | Osteoblast Function Decline in Gout |
|  | Osteoclast Activation in Gout |
|  | Neutrophil Recruitment in Sinovium in Gout |
|  | Monocytes Function in Gout |
|  | Hyper IgM Syndrome |
|  | TNF and IL1B Induce Metalloproteinase Synthesis in Osteoarthritis |
|  | Apoptosis of Chondrocyte |
|  | IL1B Induced Arthralgia |
|  | TGFB2 Signaling Impairement in Osteoarthritis |
|  | Dendritic Cell Dysfunction in Psoriatic Arthritis |
|  | Th17-Cell and Th1 Immune Responsein Psoriatic Arthritis |
|  | Osteoclast Activation in Psoriatic Arthritis |
|  | Synovial Fibroblast Activation in Psoriatic Arthritis |
|  | Keratinocyte Activation in Psoriatic Arthritis |
|  | PTPN22 Role in Rheumatoid Arthritis |
|  | Osteoclast Activation in Rheumatoid Arthritis |
|  | Synovial Fibroblast Activation by Citokines in Rheumatoid Arthritis |
|  | Apoptosis Block in Synovial Fibroblast |
|  | TLR2 Induced Synovial Fibroblast Activation in Rheumatoid Arthritis |
|  | Autocrine Cytokine/Chemokine Loops in Systemic Scleroderma |
|  | B-Cells Function in Systemic Scleroderma |
|  | Th2-Cells Function in Systemic Scleroderma |
|  | CD4+ T-Cell Function Decline in HIV |
|  | Apoptosis Block in Infected Cells |
|  | CD4+ T-Cell Death in HIV |
|  | Toll-like Receptors (TLR) in Periodontitis |
|  | Diabetes Induced Periodontitis |
|  | IL1 Receptor Antagonist (IL1RN) Deficiency (DIRA) |
|  | PAPA Syndrome |
|  | TNF Receptor Associated Periodic Syndrome |
|  | Mevalonate Kinase Deficiency |
|  | Blau Syndrome |
|  | IL36 Receptor Antagonist (IL1F5) Deficiency (DITRA) |
|  | Cryopyrin (NLPR3) Associated Periodic Syndromes |
|  | Familial Cold Autoinflammatory Syndrome 2 |
|  | Familial Mediterranean Fever |
|  | Majeed Syndrome |
|  | Gluten Impact on Neuronal System (Hypothesis) |
|  | Myostatin in Skeletal Muscles in Muscular Dystrophies |
|  | Osteoclasts Function in Osteopetrosis |
|  | Bone Loss in Osteoporosis |
|  | Hyperparathyroidism, Secondary Effect |
|  | Osteoclast Activation in Postmenopause |
|  | Microglia Activation in Alzheimer's Disease |
|  | HMGB1 and IL1B in Neuroinflammation |
|  | Microglia Activation in Parkinson's Disease |
|  | Subepithelial Fibroblasts in Middle Ear Cholesteatoma |
|  | Cholesteatoma |
|  | HPV E6 and E7 Induced Disruption of TNFR/NF-kB Signaling in Keratinocytes |
|  | OPTN Effects on NF-kB Signaling in Glaucoma |
|  | Cytokines Trigger Otitis Media |
|  | MUC5AC Expression by NTHi and S. Pneumoniae |
|  | Immune System Role in Otitis Media |
|  | Alveolar Macrophage Negative Regulation Declined in COPD |
|  | Alveolar Macrophages Dysfunction in COPD |
|  | T-Cell Cytotoxic Activation |
|  | Alveolar Epithelial Cell Dysfunction in COPD |
|  | Mucin Hyperproduction in Goblet and Mucous Cells |
|  | Endothelial Cell Dysfunction in Glomerulonephritis |
|  | Podocyte Dysfunction in Glomerulonephritis |
|  | Interstitial Fibroblasts in Pyelonephritis |
|  | Apoptosis of Renal Tubule Epithelial Cell in Pyelonephritis |
|  | Toll-like Receptors in Pyelonephritis |
|  | Endothelial Cell Dysfunction in Pyelonephritis |
|  | CD38/CD19 -> JUN/FOS/NF-kB Signaling in B-cell Proliferation |
|  | B-Cell Receptor -> NF-kB Signaling |
|  | TLR -> ARRB2/NF-kB Signaling |
|  | gamma Globulins Expression Targets |
|  | CD22 -> NF-kB Expression Targets |
|  | CD72 -> STAT Expression Target |
|  | CD8 -> STAT Expression Targets |
|  | CD80 -> STAT Expression Targets |
|  | CD86 -> STAT Expression Targets |
|  | PECAM1 Expression Targets |
|  | NFKB Canonical Signaling Activation in Cancer |
|  | NFKB Non-Canonical Signaling Activation in Cancer |
|  | STAT3 and NFkB Activate Inflammation-Induced Tumorigenesis |
|  | IL23A and IL17A Provoke Cancer-Associated Inflammation |
|  | TGFB Signaling activation by Blocking of Tumor Suppressors |
|  | NFKB Signaling Activation by Blocking of Tumor Suppressors |
|  | Apoptosis Block by Death Receptor Signaling in Cancer |
|  | Apoptosis Evasion in Cancer: Overview |
|  | Telomeric Proteins/NFKB Positive Feedback Interplay in Cancer |
|  | TGFB Family in Epithelial to Mesenchymal Transition in Cancer |
|  | IDO1 in Cancer Immune Escape |
|  | Effector T-cell Inactivation in Cancer Immune Escape |
|  | IFNLR1-> STAT/NFKB Signaling |
|  | IL1R -> NF-kB Signaling |
|  | IL16 -> NF-kB Expression Targets |
|  | PDCD1 -> NF-kB Expression Targets |
|  | DDR1 -> NF-kB Signaling |
|  | EphrinB -> MAPK/JUN/FOS Signaling |
|  | Collagen -> NF-kB Expression Targets |
|  | WNT/Calcium non-Canonical Signaling |
|  | TNF-alpha/TNFRSF1A Signaling |
|  | JNK/MAPK Signaling |
|  | TGF-beta Signaling |
|  | BMPR2 Signaling |
|  | P38 MAPK/MAPK14 Signaling |
|  | NF-kB non-Canonical Signaling |
|  | TNF-alpha/TNFRSF1B Signaling |
|  | NF-kB Canonical Signaling |
|  | TCR -> NF-kB Expression Targets |
|  | T-Cell Receptor -> AP-1 Signaling |
|  | T-Cell Receptor -> NF-kB Signaling |
|  | FASLG Expression Targets |
|  | TNF -> NF-kB Expression Targets |
|  | TNFSF10 Expression Targets |
|  | LTA Expression Targets |
|  | TNFSF14 Expression Targets |
|  | CD72 -> AP-1 Expression Targets |
|  | CD40LG -> NF-kB/ELK/SRF -> CREB/NFATC Expression Targets |
|  | TNFSF13 Expression Targets |
|  | TNFSF13B Expression Targets |
|  | EctodysplasinR -> LEF1 Signaling |
|  | TNFR -> NF-kB Signaling |
|  | TLR4 -> AP-1 Expression Targets |
|  | TLR5 Expression Targets |
|  | TLR7 Expression Targets |
|  | TLR4 -> AP-1/EGR1/HIF1A Expression Targets |
|  | TLR1 -> 2/6 Expression Targets |
|  | IL1B Expression Targets |
|  | TLR4 -> NF-kB/IRF Expression Targets |
|  | IL1A Expression Targets |
|  | TLR9 Expression Targets |
|  | TLR4/5/7/9 -> NF-kB Signaling |
|  | TLR1/2/6 -> NF-kB Signaling |
|  | TLR -> AP-1 Signaling |
|  | pathway 4 |
|  | prarg negative regulators, ps-positive |
|  | PPARG negative regulators |
|  | pparg expr target\_regulators |
|  | Neighbors of disease exacerbation |
|  | Neighbors of tumor progression |
|  | Neighbors of cancer progression |
|  | Neighbors of overall survival |
|  | Neighbors of disease severity |
|  | Neighbors of mortality |
|  | Neighbors of infarct size |
|  | Neighbors of bacterial load |
|  | Neighbors of therapeutic efficacy |
|  | Neighbors of clinical stage |
|  | Neighbors of lethality |
|  | Neighbors of tumor regression |
|  | Neighbors of macrophage count |
|  | Neighbors of body weight gain |
|  | Neighbors of neutrophil count |
|  | Neighbors of cell density |
|  | Neighbors of T-cell count |
|  | Neighbors of energy expenditure |
|  | Neighbors of radiosensitivity |
|  | Neighbors of functional status |
|  | Neighbors of infarct area |
|  | Neighbors of monocyte count |
|  | Neighbors of skin thickness |
|  | Neighbors of disease exacerbation |
|  | Neighbors of tumor progression |
|  | Neighbors of cancer progression |
|  | Neighbors of overall survival |
|  | Neighbors of disease severity |
|  | Neighbors of mortality |
|  | Neighbors of infarct size |
|  | Neighbors of bacterial load |
|  | Neighbors of therapeutic efficacy |
|  | Neighbors of clinical stage |
|  | Neighbors of lethality |
|  | Neighbors of tumor regression |
|  | Neighbors of macrophage count |
|  | Neighbors of body weight gain |
|  | Neighbors of neutrophil count |
|  | Neighbors of cell density |
|  | Neighbors of T-cell count |
|  | Neighbors of energy expenditure |
|  | Neighbors of radiosensitivity |
|  | Neighbors of functional status |
|  | Neighbors of infarct area |
|  | Neighbors of monocyte count |
|  | Neighbors of skin thickness |
|  | Neighbors of cell development |
|  | Neighbors of immune response |
|  | Neighbors of cell population |
|  | Neighbors of T-cell development |
|  | Neighbors of inflammatory response |
|  | Neighbors of wound healing |
|  | Neighbors of angiogenesis |
|  | Neighbors of innate immune response |
|  | Neighbors of cell function |
|  | Neighbors of macrophage activation |
|  | Neighbors of cell formation |
|  | Neighbors of adaptive immune response |
|  | Neighbors of osteoclast development |
|  | Neighbors of epithelial to mesenchymal transition |
|  | Neighbors of immunity |
|  | Neighbors of cell proliferative response |
|  | Neighbors of cell survival |
|  | Neighbors of osteoclast differentiation |
|  | Neighbors of cell phenotype |
|  | Neighbors of T-cell proliferation |
|  | Neighbors of cell adhesion |
|  | Neighbors of T-cell activation |
|  | Neighbors of tumor growth |
|  | Neighbors of dendritic cell development |
|  | Neighbors of cell count |
|  | Neighbors of cell interaction |
|  | Neighbors of SMC proliferation |
|  | Neighbors of endothelial cell migration |
|  | Neighbors of adipogenesis |
|  | Neighbors of lipid storage |
|  | Neighbors of cell damage |
|  | Neighbors of aging |
|  | Neighbors of cell motility |
|  | Neighbors of bone resorption |
|  | Neighbors of adipocyte differentiation |
|  | Neighbors of microglial activation |
|  | Neighbors of regeneration |
|  | Neighbors of cell migration |
|  | Neighbors of cancer growth |
|  | Neighbors of stem cell proliferation |
|  | Neighbors of hepatic regeneration |
|  | Neighbors of phagocytosis |
|  | Neighbors of immune system activation |
|  | Neighbors of osteoclast formation |
|  | Neighbors of cell invasion |
|  | Neighbors of hemopoiesis |
|  | Neighbors of bone remodeling |
|  | Neighbors of ossification |
|  | Neighbors of T-cell function |
|  | Neighbors of immune system function |
|  | Neighbors of lung development |
|  | Neighbors of autophagy |
|  | Neighbors of transcription activation |
|  | Neighbors of senescence |
|  | Neighbors of T-cell response |
|  | Neighbors of hepatocyte proliferation |
|  | Neighbors of neuroprotection |
|  | Neighbors of stem cell differentiation |
|  | Neighbors of endothelial cell function |
|  | Neighbors of neuronal death |
|  | Neighbors of cell growth |
|  | Neighbors of ROS generation |
|  | Neighbors of epithelial cell proliferation |
|  | Neighbors of cancer cell growth |
|  | Neighbors of hemato-encephalic barrier |
|  | Neighbors of cellular immune response |
|  | Neighbors of cellular senescence |
|  | Neighbors of colony formation |
|  | Neighbors of cell differentiation |
|  | Neighbors of keratinocyte proliferation |
|  | Neighbors of life span |
|  | Neighbors of cell homeostasis |
|  | Neighbors of psoriasis |
|  | Neighbors of inflammatory disease |
|  | Neighbors of rheumatoid arthritis |
|  | Neighbors of atherosclerosis |
|  | Neighbors of fibrosis |
|  | Neighbors of colitis |
|  | Neighbors of arthritis |
|  | Neighbors of inflammatory bowel disease |
|  | Neighbors of injury |
|  | Neighbors of metastasis |
|  | Neighbors of carcinogenesis |
|  | Neighbors of multiple sclerosis |
|  | Neighbors of inflammation |
|  | Neighbors of obesity |
|  | Neighbors of vascular disease |
|  | Neighbors of diabetes mellitus |
|  | Neighbors of pneumonia |
|  | Neighbors of osteoarthritis |
|  | Neighbors of liver fibrosis |
|  | Neighbors of myocardial infarction |
|  | Neighbors of leukocyte infiltration |
|  | Neighbors of airway inflammation |
|  | Neighbors of hepatitis |
|  | Neighbors of insulin resistance |
|  | Neighbors of infection |
|  | Neighbors of liver injury |
|  | Neighbors of neuroinflammation |
|  | Neighbors of death |
|  | Neighbors of encephalomyelitis |
|  | Neighbors of ischemic stroke |
|  | Neighbors of dermatitis |
|  | Neighbors of heart disease |
|  | Neighbors of diabetic nephropathy |
|  | Neighbors of immunopathology |
|  | Neighbors of hepatocellular carcinoma |
|  | Neighbors of gastric cancer |
|  | Neighbors of experimental autoimmune encephalomyelitis |
|  | Neighbors of colorectal cancer |
|  | Neighbors of cardiac remodeling |
|  | Neighbors of breast cancer |
|  | Neighbors of ischemia |
|  | Neighbors of collagen-induced arthritis |
|  | Neighbors of cartilage degeneration |
|  | Neighbors of interstitial fibrosis |
|  | Neighbors of kidney disease |
|  | Neighbors of heart failure |
|  | Neighbors of stroke |
|  | Neighbors of cancer |
|  | Neighbors of cerebral ischemia |
|  | Neighbors of neoplasm |
|  | Neighbors of atrophy |
|  | Neighbors of diet-induced obesity |
|  | Neighbors of myocardial fibrosis |
|  | Neighbors of liver disease |
|  | Neighbors of prostate cancer |
|  | Neighbors of hypertrophy |
|  | Neighbors of ovarian cancer |
|  | Neighbors of pancreatic cancer |
|  | Neighbors of proteinuria |
|  | Neighbors of lymph node |
|  | Neighbors of colon |
|  | Neighbors of gastrointestinal tract |
|  | Neighbors of central nervous system |
|  | Neighbors of Ras GTPase |
|  | Neighbors of GPCR |
|  | Neighbors of heat shock protein 90 |
|  | Neighbors of proteasome endopeptidase complex |
|  | Model of PPARG signaling in psoriasis |
|  | Model of PPARG related pathways in psoriasis (short version) |
|  | dsRNA induces innate immunity and INFs in alveolar cells |
|  | Innate immunity in Leukocytes |
|  | Dendritic Cell Activation in COVID19 |
|  | 1\_Inflammation in keratinocytes in the pathology of acne |
|  | 2\_Acute phase initiation in atopic dermatitis |
|  | 2\_Interleukin-17 and interleukin-22 signaling in psoriasis |
|  | 1\_The onset of atopic dermatitis |
|  | 1\_TNF and IL1B provoke ECM degradation in osteoarthritis |
|  | 1\_Osteoclast-mediated bone resorption in postmenopausal women |
|  | 1\_Osteoclast functional impairment in osteopetrosis |
|  | 2\_Chondrocyte death in osteoarthritis |
|  | 1\_The role of Toll-like receptors in pyelonephritis |
|  | 2\_The role of endothelial cells in pyelonephritis |
|  | 3\_The role of interstitial fibroblasts in pyelonephritis |
|  | 3\_Mesangial cell dysfunction in glomerulonephritis |
|  | 2\_Podocyte dysfunction in glomerulonephritis |
|  | 1\_Insufficient activation of immune response in the middle ear epithelium cells in otitis media |
|  | 2\_Pathogens stimulate mucins expression in the middle ear |
|  | 3\_Arterial wall inflammation and calcification in atherosclerosis |
|  | 1\_Cigarette smoke causes dysfunction and production of inflammatory mediators in alveolar epithelial cells |
|  | 3\_Role of alveolar macrophages in pulmonary tissue destruction in COPD |
|  | 1\_Th2 cell response in asthma |
|  | 3\_2\_Regulation of airway surface liquid (ASL) thickness |
|  | 3\_1\_Mucus accumulation in asthma |
|  | 2\_2\_Eosinophilia and neutrophilia in asthma: eosinophil apoptosis |
|  | 4\_Mucin hyperproduction in goblet and mucous cells in COPD |
|  | 2\_Paneth cell dysfunction in Crohn's disease |
|  | 1\_Defects in response to pathogens in the gut promotes inflammation in Crohn's disease |
|  | 3\_Autophagy dysfunction in Crohn's disease |
|  | 2\_Intestine epithelial cells drive immunological responses in ulcerative colitis |
|  | 1\_Intestine epithelial cell dysfunction in ulcerative colitis |
|  | 2\_Lipotoxicity in NAFLD |
|  | 1\_CD4+ T cell and epithelial cell response in celiac disease |
|  | 6\_p12I Viral protein promotes T-cell activation in HTLV-1 infection |
|  | 5\_Human papilloma virus E6 and E7 proteins promote epithelial cell proliferation |
|  | 2\_HIV - induced depletion of CD4+ T-cells |
|  | 4\_Inflammatory mediators inhibit GATA signaling in erythropoiesis |
|  | 4\_2\_Insulin resistance: inflammation-related insulin resistance |
|  | 4\_3\_Insulin resistance: FFA-related insulin resistance |
|  | 1\_CFTR expression and degradation in epithelial cells in cystic fibrosis (class I-II mutations) |
|  | 1\_Antigen-presenting cells promote islet dysfunction and immune system activation in T1D |
|  | 3\_Islet beta-cell destruction in T1D |
|  | 2\_Neuron and Schwann cell death |
|  | Figure 4\_Diabetes mellitus Type 1: conventional dendritic cell |
|  | Model of PPARG signaling in psoriais (tested) |
|  | Interleukin-17 and interleukin-22 signaling in psoriasis |

---

|  |  |
| --- | --- |
| MedScan ID | 6885 |
|  | 21347 |

---

|  |  |
| --- | --- |
| LocusLink ID | 6885 |
|  | 313121 |
|  | 26409 |
|  | 100910771 |
|  | 21347 |
|  | 93774 |
|  | 230048 |

---

|  |  |
| --- | --- |
| Alias | OTTHUMP00000016872 |
|  | MEKK7 |
|  | TGF-beta- activated kinase I |
|  | TGF1a |
|  | mitogen activated protein kinase kinase kinase TAK1 |
|  | TAK1 |
|  | OTTHUMP00000016870 |
|  | Transforming growth factor-beta-activated kinase 1 |
|  | TGF beta-activated kinase |
|  | mitogen-activated protein kinase kinase kinase VII |
|  | TGF-beta-activated kinase 1 |
|  | RP1-154G14.1 |
|  | mitogen-activated protein kinase kinase kinase 7 |
|  | Transforming growth factor-beta-activated kinase I |
|  | OTTMUSP00000005101 |
|  | Map3k7 |
|  | FMD2 gene |
|  | MAPKKK7 |
|  | OTTHUMP00000016871 |
|  | RP23-447J12.1 |
|  | OTTMUSP00000005100 |
|  | OTTHUMP00000016873 |
|  | CSCF |
|  | FMD2 |
|  | TGF-beta activated kinase 1 |
|  | mitogen activated protein kinase kinase kinase 7 |
|  | B430101B05 |
|  | LOC100910771 |
|  | EST C87327 |
|  | mitogen-activated protein kinase kinase kinase 7-like |
|  | transforming growth factor beta-activated kinase 1 |
|  | C87327 |
|  | TGF-beta- activated kinase 1 |
|  | mitogen activated protein kinase kinase kinase 7 (predicted) |

---

|  |  |
| --- | --- |
| EC Number | 2.7.11.25 |
|  | 2.7.1.- |

---

|  |  |
| --- | --- |
| GO ID | 0005524 |
|  | 0004709 |
|  | 0000287 |
|  | 0004672 |
|  | 0004674 |
|  | 0030971 |
|  | 0097110 |
|  | 0038095 |
|  | 0007249 |
|  | 0007252 |
|  | 0007254 |
|  | 0002755 |
|  | 0050852 |
|  | 0007223 |
|  | 0000187 |
|  | 0000186 |
|  | 0007250 |
|  | 0043276 |
|  | 0043966 |
|  | 0070498 |
|  | 0070423 |
|  | 0043123 |
|  | 0043507 |
|  | 0051092 |
|  | 0002726 |
|  | 0032743 |
|  | 0016239 |
|  | 0016579 |
|  | 0002223 |
|  | 0051403 |
|  | 0007179 |
|  | 0016032 |
|  | 0005671 |
|  | 0008385 |
|  | 0005829 |
|  | 0010008 |
|  | 0005634 |
|  | 0005886 |
|  | 0004708 |
|  | 0000165 |
|  | 0001525 |
|  | 0006915 |
|  | 2001234 |
|  | 0060546 |
|  | 2000378 |
|  | 1902443 |
|  | 0001841 |
|  | 0018107 |
|  | 0046330 |
|  | 0006468 |
|  | 2000377 |
|  | 0005737 |
|  | 0014069 |
|  | 0005622 |
|  | 0050870 |
|  | 0045087 |
|  | 0034146 |
|  | 0035872 |
|  | 0016310 |
|  | 0034142 |
|  | 0006351 |
|  | 0007165 |
|  | 0002756 |
|  | 0043066 |
|  | 0006355 |
|  | 0097481 |
|  | 0006950 |
|  | 0005515 |
|  | 0034134 |
|  | 0034138 |
|  | 0035666 |
|  | 0008063 |
|  | 0034130 |
|  | 0034166 |
|  | 0038123 |
|  | 0038124 |
|  | 0016772 |
|  | 0034162 |
|  | 0046872 |
|  | 0005654 |
|  | 0016301 |
|  | 0016740 |
|  | 0016020 |
|  | 0002224 |
|  | 0000166 |
|  | 0008152 |
|  | 0004713 |

---

|  |  |
| --- | --- |
| KEGG ID | hsa:6885 |
|  | rno:100910771 |
|  | rno:313121 |
|  | mmu:26409 |

---

|  |  |
| --- | --- |
| MedLine Reference | 12969270 |
|  | 15831522 |
|  | 16157589 |
|  | 16183734 |
|  | 17085580 |
|  | 10349636 |
|  | 10725249 |
|  | 10757786 |
|  | 10922068 |
|  | 11042159 |
|  | 11076861 |
|  | 11217851 |
|  | 11356033 |
|  | 12464436 |
|  | 12466851 |
|  | 12477932 |
|  | 12545162 |
|  | 12556533 |
|  | 12711537 |
|  | 12805214 |
|  | 12842894 |
|  | 14592977 |
|  | 15226265 |
|  | 15647289 |
|  | 15782199 |
|  | 15837794 |
|  | 15894542 |
|  | 15911626 |
|  | 16141072 |
|  | 16141073 |
|  | 16145668 |
|  | 16186825 |
|  | 16260493 |
|  | 16301747 |
|  | 16339560 |
|  | 16360132 |
|  | 16385569 |
|  | 16446357 |
|  | 16498455 |
|  | 16556914 |
|  | 16675448 |
|  | 16754690 |
|  | 16799562 |
|  | 16831874 |
|  | 16835226 |
|  | 16857737 |
|  | 16919269 |
|  | 16940043 |
|  | 8533096 |
|  | 10022904 |
|  | 10094049 |
|  | 10187861 |
|  | 10195894 |
|  | 10224067 |
|  | 10748100 |
|  | 10779355 |
|  | 10807933 |
|  | 10838074 |
|  | 10882101 |
|  | 10921914 |
|  | 11104763 |
|  | 11118615 |
|  | 11259596 |
|  | 11397816 |
|  | 11460167 |
|  | 11585904 |
|  | 11809792 |
|  | 11865055 |
|  | 12048196 |
|  | 12089335 |
|  | 12242293 |
|  | 12296995 |
|  | 12372426 |
|  | 12547194 |
|  | 12589052 |
|  | 12609980 |
|  | 12804775 |
|  | 12859960 |
|  | 12874243 |
|  | 14702039 |
|  | 14743216 |
|  | 14960582 |
|  | 15075345 |
|  | 15082531 |
|  | 15125833 |
|  | 15277532 |
|  | 15590691 |
|  | 15670770 |
|  | 15764709 |
|  | 15917296 |
|  | 16000313 |
|  | 16153868 |
|  | 16251197 |
|  | 16260783 |
|  | 16280329 |
|  | 16293250 |
|  | 16527194 |
|  | 16893890 |
|  | 17114179 |
|  | 3031469 |
|  | 7708055 |
|  | 8638164 |
|  | 9278437 |
|  | 9380693 |
|  | 9466656 |
|  | 9480845 |
|  | 9556573 |
|  | 9689078 |
|  | 14574404 |
|  | 15489334 |
|  | 16289117 |
|  | 17081983 |
|  | 17158449 |
|  | 17173073 |
|  | 17202147 |
|  | 17276978 |
|  | 17379600 |
|  | 17114649 |
|  | 17197697 |
|  | 17209006 |

---

|  |  |
| --- | --- |
| Organism | Homo sapiens {Organism urn:agi-taxid:9606} |
|  | Rattus norvegicus {Organism urn:agi-taxid:10116} |
|  | Homo sapiens |
|  | Rattus norvegicus |
|  | Mus musculus |

---

|  |  |
| --- | --- |
| Mouse chromosome position | 4 |
|  | 4 A5 |

---

|  |  |
| --- | --- |
| OMIM ID | 602614 |
|  | 157800 |
|  | 617137 |
|  | OMIM:602614 |

---

|  |  |
| --- | --- |
| Rat chromosome position | 5q21 |
|  | 9 |

---

|  |  |
| --- | --- |
| Hugo ID | 6859 |
|  | HGNC:6859 |

---

|  |  |
| --- | --- |
| Human chromosome position | 6q15 |
|  | 6q16.1-q16.3 |

---

|  |  |
| --- | --- |
| Swiss-Prot Accession | O43318 |
|  | O43318.1 |
|  | P0C8E4 |
|  | P0C8E4.1 |
|  | B2RE27 |
|  | E1P523 |
|  | O43317 |
|  | O43319 |
|  | Q5TDN2 |
|  | Q5TDN3 |
|  | Q5TDT7 |
|  | Q9NTR3 |
|  | Q9NZ70 |
|  | Q543B5 |
|  | Q923A8 |
|  | Q62073 |
|  | Q5U0C9 |
|  | Q5U0D0 |
|  | Q9UG54 |
|  | Q3TXG1 |
|  | Q5TDM9 |
|  | M3K7\_HUMAN |

---

|  |  |
| --- | --- |
| PIR ID | JC5955 |
|  | JC5956 |

---

|  |  |
| --- | --- |
| GenBank ID | NC\_000006 |
|  | NM\_145333 |
|  | NP\_663306 |
|  | NM\_003188 |
|  | NP\_003179 |
|  | NM\_145332 |
|  | NP\_663305 |
|  | NM\_145331 |
|  | NP\_663304 |
|  | XM\_006715553 |
|  | XP\_006715616 |
|  | XM\_017011226 |
|  | XP\_016866715 |
|  | NG\_011966 |
|  | AL121837 |
|  | AL121964 |
|  | CH471051 |
|  | EAW48525 |
|  | EAW48526 |
|  | EAW48527 |
|  | EAW48528 |
|  | EAW48529 |
|  | DQ314875 |
|  | ABC40734 |
|  | AB009356 |
|  | BAA25025 |
|  | AB009357 |
|  | BAA25026 |
|  | AB009358 |
|  | BAA25027 |
|  | AF218074 |
|  | AAF27652 |
|  | AI147171 |
|  | AK055901 |
|  | AK299268 |
|  | BAG61290 |
|  | AK315774 |
|  | BAG38124 |
|  | AL050393 |
|  | CAB43687 |
|  | AL520975 |
|  | BC017715 |
|  | AAH17715 |
|  | BM172351 |
|  | BT019654 |
|  | AAV38460 |
|  | BT019655 |
|  | AAV38461 |
|  | BX648277 |
|  | O43318 |
|  | NC\_005104 |
|  | XM\_006237966 |
|  | XP\_006238028 |
|  | NM\_001107920 |
|  | NP\_001101390 |
|  | AC\_000073 |
|  | AABR07047779 |
|  | AAHX01035853 |
|  | AAHX01035854 |
|  | CH473962 |
|  | EDL98558 |
|  | EDL98559 |
|  | BC099193 |
|  | P0C8E4 |
|  | NP\_033342 |
|  | CAI19609 |
|  | XM\_003750753 |
|  | AK046565 |
|  | BAA11184 |
|  | XP\_006245676 |
|  | CAI19610 |
|  | CAI19611 |
|  | XP\_006538011 |
|  | CAI19612 |
|  | CAI19613 |
|  | NM\_172688 |
|  | AL833781 |
|  | AAHY01034689 |
|  | ABBA01040553 |
|  | ABBA01040554 |
|  | BAC35588 |
|  | Q62073 |
|  | BAE34955 |
|  | AK145302 |
|  | XM\_006537948 |
|  | AC\_000026 |
|  | CAI23532 |
|  | CAI23533 |
|  | CAI23530 |
|  | CAI23531 |
|  | CV559615 |
|  | NC\_000070 |
|  | AC\_000138 |
|  | CAM24620 |
|  | NP\_766276 |
|  | CAM24622 |
|  | CAM24621 |
|  | CAM24623 |
|  | NM\_009316 |
|  | XR\_390323 |
|  | D76446 |
|  | XP\_005248818 |
|  | AK211827 |
|  | NC\_005108 |
|  | XM\_006245614 |
|  | AAHY01034690 |
|  | AAH06665 |
|  | AK053911 |
|  | AK137046 |
|  | EDL05512 |
|  | XP\_003750801 |
|  | EDL05511 |
|  | AK159277 |
|  | BAE23216 |
|  | AK133642 |
|  | NC\_018917 |
|  | AABR07068650 |
|  | AMYH02014101 |
|  | BC006665 |
|  | CH466538 |
|  | AABR06036336 |
|  | XM\_005248761 |
|  | NT\_007299 |
|  | NW\_923184 |
|  | NW\_001838987 |
|  | AC\_000049 |
|  | CR604679 |
|  | Q5U0C9 |
|  | Q5U0D0 |
|  | Q9NZ70 |
|  | Q9UG54 |
|  | NT\_166289 |
|  | NW\_001030740 |
|  | Q3TXG1 |
|  | Q543B5 |
|  | Q923A8 |
|  | NW\_047711 |
|  | NW\_001084836 |
|  | XM\_232855 |
|  | XP\_232855 |
|  | XM\_001062961 |
|  | XP\_001062961 |
|  | Q5TDM9 |
|  | BG067741 |
|  | BG085694 |
|  | NT\_086697 |
|  | Q5TDN2 |
|  | Q5TDN3 |
|  | NW\_000206 |
|  | NT\_109315 |
|  | BG080801 |
|  | 10720099 |
|  | 1167506 |
|  | 123787232 |
|  | 13879376 |
|  | 26343863 |
|  | 74192894 |
|  | 74205839 |
|  | 81909144 |
|  | 81916394 |
|  | C87327 |
|  | 119568910 |
|  | 119568911 |
|  | 119568912 |
|  | 119568913 |
|  | 119568914 |
|  | 12643557 |
|  | 17389343 |
|  | 2924624 |
|  | 2924626 |
|  | 54696176 |
|  | 54696178 |
|  | 56203689 |
|  | 56203690 |
|  | 56203691 |
|  | 56203692 |
|  | 56203693 |
|  | 56204060 |
|  | 56204061 |
|  | 56204062 |
|  | 56204063 |
|  | 6562171 |
|  | 6746615 |
|  | 74753307 |
|  | 74756530 |
|  | 74756708 |
|  | 74756709 |
|  | 74761825 |
|  | 83699657 |
|  | 8978252 |
|  | 123230057 |
|  | 123230058 |
|  | 123230059 |
|  | 123230060 |
|  | 148673564 |
|  | 148673565 |
|  | 149045558 |
|  | 149045559 |

---

|  |  |
| --- | --- |
| Swiss-Prot ID | M3K7\_HUMAN |
|  | M3K7\_MOUSE |
|  | M3K7\_RAT |
|  | Q9NZ70\_HUMAN |
|  | Q923A8\_MOUSE |
|  | Q543B5\_MOUSE |
|  | B2RE27\_HUMAN |
|  | Q5TDT7\_HUMAN |

---

|  |  |
| --- | --- |
| Cell Localization | Cytoplasm |
|  | Cell membrane |

---

|  |  |
| --- | --- |
| Ensembl ID | ENSG00000135341 |
|  | ENSP00000358338.3 |
|  | ENST00000369332.7 |
|  | ENSP00000358335.3 |
|  | ENST00000369329.8 |
|  | ENSP00000358331.3 |
|  | ENST00000369325.7 |
|  | ENSP00000358333.3 |
|  | ENST00000369327.7 |
|  | ENSRNOG00000005724 |
|  | ENSRNOP00000007654.4 |
|  | ENSRNOT00000007654.5 |
|  | ENSMUSG00000028284 |
|  | ENSP00000358331 |
|  | ENSP00000358333 |
|  | ENSP00000358335 |
|  | ENSP00000358338 |
|  | ENST00000369325 |
|  | ENSMUST00000037607 |
|  | ENSMUST00000080933 |
|  | ENSRNOP00000007654 |
|  | ENSRNOP00000007657 |
|  | ENSRNOT00000007654 |
|  | ENST00000369329 |
|  | ENST00000369327 |
|  | ENST00000369329.7 |
|  | ENSMUSP00000079734 |
|  | ENST00000369332 |
|  | ENSMUSP00000040307 |
|  | ENSRNOG00000047516 |

---

|  |  |
| --- | --- |
| MGI ID | MGI:1346877 |
|  | 1346877 |
|  | 2135559 |
|  | 1203726 |

---

|  |  |
| --- | --- |
| RGD ID | 1309438 |
|  | 6496502 |

---

|  |  |
| --- | --- |
| Unigene ID | Hs.594838 |
|  | Rn.24019 |
|  | Mm.258589 |
|  | Hs.722892 |
|  | Hs.644143 |
|  | Mm.392292 |
|  | Hs.290346 |
|  | Rn.100643 |
|  | Hs.485968 |
|  | Hs.652105 |

---

|  |  |
| --- | --- |
| Homologene ID | 37743 |
|  | 135715 |
|  | 118818 |

---

|  |  |
| --- | --- |
| Shape | Sickle-vertex |

---

|  |  |
| --- | --- |
| IPI ID | IPI00121080 |
|  | IPI00218566 |
|  | IPI00218567 |
|  | IPI00295738 |
|  | IPI00367017 |
|  | IPI00241935 |
|  | IPI00123967 |
|  | IPI00641808 |
|  | IPI00648040 |
|  | IPI00649898 |
|  | IPI00214313 |

---

|  |  |
| --- | --- |
| Homo sapiens Chromosome position | 6q16.1-q16.3 |

---

|  |  |
| --- | --- |
| Ensembl Transcript ID | ENST00000369332 |
|  | ENST00000369329 |
|  | ENST00000369325 |
|  | ENST00000369327 |

---

|  |  |
| --- | --- |
| FunctionalClass | MAP kinase kinase kinase |
|  | protein binding |
|  | protein-tyrosine kinase |
|  | ATP binding |
|  | kinase |
|  | magnesium ion binding |
|  | metal ion binding |
|  | nucleotide binding |
|  | protein kinase |
|  | protein serine/threonine kinase |
|  | transferase |
|  | MAP kinase kinase |

---

|  |  |
| --- | --- |
| Mus musculus Chromosome position | 4 A5 |
|  | 4 |

---

|  |  |
| --- | --- |
| Rattus norvegicus Chromosome position | 5q21 |

---

|  |  |
| --- | --- |
| Ensembl Gene ID | ENSG00000135341 |

---
